# Nutrigenomic evidence of phytogenic cytoprotective functions in the ovary and liver provides mechanistic support for improved laying hen performance

**DOI:** 10.1080/10495398.2025.2463995

**Published:** 2025-02-13

**Authors:** Ioannis Brouklogiannis, Konstantinos C. Mountzouris

**Affiliations:** Laboratory of Nutritional Physiology and Feeding, Department of Animal Science, Agricultural University of Athens, Athens, Greece

**Keywords:** Poultry, phytochemical, gene expression, AhR, Nrf2

## Abstract

The study assessed the inclusion level effects of a phytogenic blend (PB) on performance and critical molecular biomarkers related to detoxification (Aryl hydrocarbon receptor; AhR) and antioxidant (Nuclear factor-erythroid 2-related factor 2; Nrf2) responses in layers’ ovary and liver. Layers (n = 385; 21-week-old; Hy-Line Brown) were allotted to 5 treatments with 7 replicates of 11 hens each, for a 12-week feeding trial. Treatments were: control (CON) without PB or supplementation with PB at 250 (PB250), 750 (PB750), 1000 (PB1000) and 1500 mg/kg diet (PB1500), respectively. Performance was determined weekly for the 12-week experimental period. At the 6^th^ and 12^th^ experimental week ovarian and liver samples were collected for gene expression analysis. Increasing PB inclusion level, improved linearly and quadratically overall laying rate, egg mass and FCR, with optimal (*P* ≤ 0.001) performance notable in the PB750 compared to CON. The nutrigenomic analysis revealed that PB inclusion resulted (*P* ≤ 0.05) in consistent beneficial modulation of the AhR/Nrf2 pathway-related genes assessed at the 6^th^ and 12^th^ experimental week, both in the ovary and the liver of laying hens. In conclusion, PB beneficially modulated the ovarian and hepatic adaptive cytoprotection and supported the laying performance improvements recorded, with PB750 displaying the optimal benefits.

## Introduction

It is known that the ovary and liver, play essential roles in various aspects of hen’s overall health and productive performance.^1^^,^^2^ In particular, the ovary represents a crucial organ for laying and breeder hens, that is tightly linked with the re-productive performance. In addition, the ovary produces hormones with an indirect influence on liver function. On the other hand, the liver besides its key role in metabolic functions, is also central to regulating ovarian follicle development by metabolizing hormones (such as estrogen and progesterone) and managing nutrient levels (such as glucose and lipids), both of which support ovarian development and reproductive health.^2^^,^^3^ Subsequently, ovarian and/or hepatic compromised homeostatic function is accompanied by reproductive and metabolic disorders, as well as reduced persistency of lay and productivity of the layers.

In commercial poultry farming, stressor challenges regardless of their source or type (e.g., nutritional, environmental or biological), may lead to excessive production of reactive oxygen species (ROS) and subsequent oxidative stress. The latter is ultimately associated with aging and fatty liver syndrome, contributing to ovarian and liver dysfunction, respectively.^1^^,^^4–6^

In human nutrition, the Mediterranean diet has been the subject of much interest because of its association with human longevity and well-being.^7^^,^^8^ The protective role of the Mediterranean diet is attributed to bioactive compounds with various therapeutic and overall health-promoting properties. Native plants, aromatic herbs and spices represent essential ingredients of the Mediterranean diet.^9^^,^^10^ In this sense, plant-based bioactive compounds represent a credible tool to control and manage the generation of ROS and subsequent oxidative damage. In particular, phytogenics including Mediterranean indigenous plants (olive and citrus trees) and herbs (oregano and thymus) contain bioactive substances that could mitigate oxidative stress and enhance the productive performance of poultry due to their cytoprotective functions.^11–13^ The cytoprotection mediated by dietary phytogenics could be via the modulation of two crucial signaling pathways: the aryl hydrocarbon receptor (AhR) responsible for detoxification and the nuclear factor erythroid 2-related factor 2 (Nrf2) responsible for the adaptive antioxidant defense.^14–16^ Both AhR/Nrf2 are essential components for the host overall cytoprotection and homeostasis.^16^

Given all the above, the topic of AhR/Nrf2 modulation and the respective cytoprotective potential in layers’ ovary and liver receives current scientific attention.^6^^,^^17^ Research evidence with poultry demonstrates that phytogenic efficacy may depend on phytogenic composition and inclusion level in the diet^6^^,^^18^ as well as on the production phase of administration.^19^^,^^20^ In layers, the peak production phase represents a period of paramount importance not only due to the intense daily ovulations and high productivity but also for conditioning layers for prolonged production across their lifespan.^19^^,^^21^ However, in addition to open questions with regard to the efficacy of various phytogenics, there is an overall lack of information on phytogenic applications in the peak production phase as most of the existing studies have focused on the post-peak phase.^17^^,^^21^^,^^22^

Therefore, this study focused on the peak production phase (22–33 week old) of laying hens and aimed to generate new information on the inclusion level effects of a Mediterranean plants-based dietary phytogenic blend on layer productive performance and the adaptive cytoprotective response at a molecular level. In particular, via a nutrigenomic approach, the expression of critical genes relevant to the detoxification (AhR pathway) and antioxidant (Nrf2 pathway) responses was assessed in the ovary and liver.

## Materials and methods

### Ethical statement

All the procedures and practices used in the current study regarding the care and use of animals for research purposes were under the institutional and national guidelines and approved by the Bioethics Committee of the Agricultural University of Athens (AUA), Greece (Protocol number: 16/12012022).

### Birds, diets, and experimental design

In total, 385 Hy-Line Brown laying hens were reared in the AUA laying hen facility for a 6-week adaptation period. At the end of the 21^st^ week of age, layers were allotted into 5 dietary treatments with 7 replicates of 11 layers each, having uniform body weight and similar performance. The experimental trial was carried out between 22 and 33 week of layers age (i.e., 12 week experimental period). Depending on the phytogenic blend (PB) inclusion level (0, 250, 750, 1000 and 1500 mg/kg) in the basal diet, treatments were: CON, PB250, PB750, PB1000 and PB1500, respectively. The PB consisted of selected Mediterranean plants having olive oil polyphenols, carvacrol and thymol among its main bioactive components with a total bioactive content of 50 g/kg PB (nuPhoria^®^; Nuevo SA, Greece). All experimental treatments received a maize-soybean meal basal diet in mash form formulated to meet or exceed the recommendations according to the Hy-Line brown Management Guide (2018). The basal diet’s composition is shown in Table 1. Experimental diets and water were available *ad libitum*. The laying hens were housed in 3-floor battery cages (192 cm length × 45 cm width × 48 cm height) under controlled environmental conditions, and gradually increasing light photoperiod, to reach 16 hours of light (16 L:8D) at approximately 30 weeks of age, according to the Light Program for Light provided in Hy-line brown Management Guide (2018).

**Table 1.** Ingredient and chemical composition of the basal experimental diet (%).

	%
Ingredients	
Corn grain	52.23
Soybean meal (44%)	23.66
Soy Protein concentrate^1^	2.82
Soya oil	3.00
Vegetable fat^2^	3.00
Wheat bran	3.00
Limestone	9.76
Monocal phosphate, HCL	1.58
Salt (NaCl)	0.30
Sodium bicarbonate	0.13
L-lysine-HCL	0.05
DL-methionine	0.31
L-threonine	0.06
Vitamin + mineral premix^3^	0.10
Chemical composition^4^	
AME_n_, (MJ/kg diet)	12.17
Dry matter	90.96
Crude protein	16.50 (16.51)
Ether extract	8.05 (7.95)
Crude fiber	3.23
Calcium	4.10
Available phosphorus	0.48
Lysine	0.92
TSAA^5^ (methionine + cysteine)	0.86
Threonine	0.69

^1^Soy Protein concentrate with 530 g crude protein/kg (Alpha Soy 530, Agilia Europe, Skjernvej 42, DK-6920, Videbaek, Denmark).

^2^Lecithinised fat powder with 6% lecithin (nufat 99 L, Nuevo SA, Schimatari, Viotia, Greece).

^3^Vitamin + mineral premix (Nuevo SA, Schimatari, Viotia, Greece) provided the following per kg of diet: vitamin A, 8000 IU; vitamin D3, 3200 IU; vitamin E, 20 mg; vitamin K (menadione), 2.5 mg; vitamin B1 (thiamin), 2.5 mg; vitamin B2 (riboflavin), 5.5 mg; vitamin B3 (niacin), 30 mg; vitamin B5 (pantothenic acid), 8 mg; vitamin B6 (pyridoxine), 4 mg; vitamin B7 (biotin), 0.075 mg; vitamin B9 (folic acid), 0.9 mg; vitamin B12 (cobalamine), 0.023 mg; manganese, 90 mg; zinc, 80 mg; iron, 40 mg; copper, 8 mg; iodine, 1.2 mg and selenium, 0.22 mg.

^4^The values in parentheses were analyzed, and others were calculated.

^5^Total sulfuric amino acids.

### Determination of productive performance

Throughout the study, the total number of eggs produced and total egg weight were recorded daily. The laying rate was determined on a weekly basis as the total number of eggs divided by 7 days. The average egg weight was determined as the total egg weight divided by the number of eggs produced in 7 days. Furthermore, every week, the feed intake of laying hens was recorded. Egg mass was calculated by multiplying the average egg weight by the laying rate. Feed conversion ratio was calculated as grams of feed intake per gram of egg mass. The data for the performance responses (i.e., laying rate, egg mass, feed intake and feed conversion ratio) were reported as overall (1–12^th^ experimental week). Mortality data were recorded daily throughout the experimental period.

### Sample collection

At the end of the 6^th^ and 12^th^ experimental week (i.e., 27 and 33 week of layers age), 7 layers per treatment were randomly selected (1 layer per replicate), anesthetized (EC 1099/2009) and euthanized by severing the jugular vein. Afterward, ovarian follicles and liver samples were carefully excised aseptically, snap-frozen in liquid nitrogen, and subsequently stored at −80 °C for further analyses.

### RNA isolation and reverse transcription to cDNA

For the determination of target gene expression, total RNA was extracted from ovarian and liver samples employing the NucleoZOL Reagent (Macherey-Nagel GmbH & Co. KG, Düren, Germany), according to the manufacturer’s guidelines. Subsequently, RNA quantity and quality were assessed spectrophotometrically with the use of NanoDrop-1000 (Thermo Fisher Scientific, Waltham, United Kingdom) followed by DNase treatment. In particular, 10 μg of RNA were diluted with 1 U of DNase I (M0303, New England Biolabs Inc, Ipswich, UK) and 10 μL of 10x DNase buffer to a final volume of 100 µL with the addition of DEPC water for 20 min at 37 °C. Before DNase inactivation at 75 °C for 10 min, 1 μL of 0.5 mol/L EDTA was added to protect RNA from being degraded during enzyme inactivation. RNA integrity was evaluated by agarose gel electrophoresis. For cDNA preparation, 500 ng of total RNA from each sample was reverse transcribed to cDNA by PrimeScript RT Reagent Kit (Perfect Real Time, Takara Bio Inc., Shiga-Ken, Japan) following the manufacturer’s recommendations. The final cDNA solutions were stored at −20 °C for further use.

### Quantitative Real-Time PCR

The following *Gallus gallus* genes were assessed: aryl hydrocarbon receptor 1 (*Ahr1*), aryl hydrocarbon receptor nuclear translocator (*ARNT*), cytochrome P450 1A1 (*CYP1A1*), cytochrome P450 1A2 (*CYP1A2*), cytochrome P450 1B1 (*CYP1B1*), nuclear factor erythroid-derived 2-like 2 (Nrf2), kelch like ECH associated protein 1 (*Keap1*), catalase (*CAT*), superoxide dismutase 1 (*SOD1*), glutathione peroxidase 2 (*GPX2*), glutathione S-transferase alpha 2 (*GSTA2*), NAD(P)H quinone dehydrogenase 1 (*NQO1*) and heme oxygenase 1 (*HMOX1*). Suitable primers were designed using the GenBank sequences deposited on the National Center for Biotechnology Information and US National Library of Medicine (NCBI) are shown in Table 2. The primers were checked using the PRIMER BLAST algorithm for *Gallus gallus* mRNA databases to ensure that there was a unique amplicon. Real-time quantitative PCR (qPCR) was performed in 96-well microplates with a SaCycler-96 Real-Time PCR System (Sacace Biotechnologies s.r.l., Como, Italy) and FastGene IC Green 2x qPCR universal mix (Nippon Genetics, Tokyo, Japan). Each reaction included 12.5 ng RNA equivalents along with 200 nmol/L of forward and reverse primers for each gene. The qPCR conditions were as follows: initial heating to 95 °C for 3 min, accompanied by 40 cycles of 95 °C for 5 s, 59.5 to 62 °C (depending on the target gene) for 20 s and 72 °C for 33 s. This was tailed by a melt curve analysis to confirm the specificity of the reaction. Every sample was measured in duplicates. Relative gene expression ratios were calculated according to,^23^ adapted for the multi-reference genes normalization procedure according to^24^ using glyceraldehyde 3-phosphate dehydrogenase (*GAPDH)* and beta (β)-actin (*ACTB)* as housekeeping genes.

**Table 2.** Primer sequences used in this work for quantitative real-time PCR.

Gene symbol^1^	Primer sequence (5′-3′)^2^	Annealing temperature (⁰C)	PCR product size (bp)	GenBank (NCBI reference sequence)
*GAPDH*	F: ACTTTGGCATTGTGGAGGGT R: GGACGCTGGGATGATGTTCT	59.5	131	NM_204305.1
*ACTB*	F: CACAGATCATGTTTGAGACCTT R: CATCACAATACCAGTGGTACG	60	101	NM_205518.1
AhR pathway				
*AhR1*	F: TTTAGTGTGGCAGGTGGATT R: CCTTGTGCCAATGATGCTATTTG	60	200	NM_204118.2
*ARNT*	F: GAGACCAAGGCCCCAACTAC R: TCGGGTGCCTCTTTCTTTCC	62	140	NM_204200.1
*CYP1A1*	F: GTGATGGAGGTGACCATCGG R: ACATTCGTAGCTGAACGCCA	62	165	NM_205147.1
*CYP1A2*	F: CTGACCGTACACCACGCTT R: CTCGCCTGCACCATCACTTC	62	75	NM_205146.2
*CYP1B1*	F: CAGTGACTCCGCATCCCAAA R: CCATACGCTTACGGCAGGTT	62	132	XM_015283751.2
Nrf2 pathway				
*Nrf2*	F: AGACGCTTTCTTCAGGGGTAG R: AAAAACTTCACGCCTTGCCC	60	285	NM_205117.1
*Keap1*	F: GGTTACGATGGGACGGATCA R: CACGTAGATCTTGCCCTGGT	62	135	XM_025145847.1
*CAT*	F: ACCAAGTACTGCAAGGCGAA R: TGAGGGTTCCTCTTCTGGCT	60	245	NM_001031215
*SOD1*	F: AGGGGGTCATCCACTTCC R: CCCATTTGTGTTGTCTCCAA	60	122	NM_205064.1
*GPX2*	F: GAGCCCAACTTCACCCTGTT R: CTTCAGGTAGGCGAAGACGG	62	75	NM_001277854.1
*GSTA2*	F: GCCTGACTTCAGTCCTTGGT R: CCACCGAATTGACTCCATCT	60	138	NM_001001776.1
*NQO1*	F: GAGCGAAGTTCAGCCCAGT R: ATGGCGTGGTTGAAAGAGGT	60.5	150	NM_001277619.1
*HMOX1*	F: ACACCCGCTATTTGGGAGAC R: GAACTTGGTGGCGTTGGAGA	62	134	NM_205344.1

^1^GAPDH = glyceraldehyde 3-phosphate dehydrogenase; ACTB = actin beta; AhR1 = aryl hydrocarbon receptor 1; ARNT = aryl hydrocarbon receptor nuclear translocator; CYP1A1 = cytochrome P450 1A1; CYP1A2 = cytochrome P450 1A2; CYP1B1 = cytochrome P450 1B1; Nrf2 = nuclear factor; erythroid 2-like 2; Keap1 = kelch-like ECH-associated protein 1; CAT = catalase; SOD1 = superoxide dismutase 1; GPX2 = glutathione peroxidase 2; GSTA2 = glutathione S-transferase alpha 2; NQO1 = NAD(P)H quinone dehydrogenase 1; HMOX1 = heme oxygenase1.

^2^F: Forward, R: Reverse.

### Statistical analysis

Experimental data on layer productive performance were based on a cage basis, whereas ovarian and liver gene expression ratios were based on individual layers. All data were tested for normality using the Kolmogorov-Smirnov test and found to be normally distributed. Subsequently, data were analyzed with the general linear model (GLM)—ANOVA procedure using the SPSS for Windows statistical package program, version 27 (SPSS Inc., Chicago, IL). Statistically significant effects were further analyzed, and means were compared using Tukey’s honest significant difference (HSD) multiple comparison procedure. Statistical significance was determined at *P* ≤ 0.05. Linear (lin) and quadratic (quad) response patterns to dietary PB inclusion level were studied using polynomial contrasts.

## Results

### Productive performance

The effects of PB inclusion level on the overall productive performance responses of laying hens are shown in Table 3. Compared to the non-supplemented control group, dietary inclusion of PB led to significant (*P* ≤ 0.001) improvements in laying rate, egg mass and feed conversion ratio for the overall period (1–12 experimental week, i.e., 21–33 week of layers age). There were no (*P* > 0.05) differences between the experimental treatments for the overall feed intake and egg weight. Overall laying rate increased in a linear (*P_lin_* = 0.031) and quadratic (*P_quad_* < 0.001) manner with increasing PB inclusion level with PB250, PB750 and P1000 being significantly higher (P < 0.001) than CON. Overall egg mass displayed a linear (*P_lin_* = 0.028) and a quadratic (*P_quad_* < 0.001) pattern of increase with increasing PB inclusion level. More specifically, treatments PB750 and PB1000 had higher (*P* < 0.001) egg mass than the control, with PB250 and PB1500 being intermediate and not different from the other three treatments. In addition, increasing PB inclusion level resulted in linear (*P_lin_* < 0.021) and quadratic (*P_quad_* < 0.001) patterns of decrease for feed conversion ratio with PB250, PB750 and P1000 being higher (*P* < 0.001) than CON. More specifically, laying rate, egg mass and feed conversion ratio were better primarily in treatment PB750 and secondarily in PB1000 compared to the control. Mortality throughout the experiment was zero.

**Table 3.** Effects of the dietary phytogenic blend inclusion level on the overall laying productive performance.

Responses	Treatments^1^						Statistics^2^		
(1 to 12 week^4^)	CON	PB250	PB750	PB1000	PB1500	SEM^3^	*P_anova_*	*P_linear_*	*P_quadratic_*
Laying Rate %	94.8^b^	96.7^a^	98.0^a^	97.6^a^	96.2^ab^	0.66	<0.001	0.031	<0.001
Egg mass g/hen/d	58.3^b^	59.8^ab^	60.8^a^	60.7^a^	59.4^ab^	0.58	<0.001	0.028	<0.001
Feed intake g/hen/d	116.6	116.4	116.6	117.0	116.3	0.86	0.909	0.989	0.989
Feed conversion ratio	2.00^a^	1.95^b^	1.92^b^	1.93^b^	1.96^ab^	0.017	0.001	0.021	<0.001
Egg weight g	61.5	61.8	62.0	62.2	61.8	0.38	0.533	0.307	0.212

^1^PB Inclusion (CON = 0 mg/kg, PB250 = 250mg/kg, PB750 = 750mg/kg, PB1000 = 1000mg/kg and PB1500 = 1500mg/kg of diet). Data represent treatment means based on 11 hens per replicate and 7 replicates per treatment (*n* = 7).

^2^Means with no common superscripts (a, b) within the same row differ significantly (*P* < 0.05).

^3^Standard error of the means.

^4^Reference to 1–12 week of overall experimental period corresponds to 22–33 week of layers age.

### mRNA transcript levels of the AhR pathway genes in the ovary at the 6th and 12th week

In the ovary, the relative expression levels of the *AhR* pathway (*AhR1*, *ARNT*, *CYP1A1*, *CYP1A2* and *CYP1B1*) related genes at week 6 and week 12 (i.e., 27 and 33 week of layers age) are presented in Table 4. In the ovary, there were no significant (*P* > 0.05) differences between the experimental treatments for any of the genes studied at week 6 of the experiment (27^th^ week of layers age). Polynomial contrast analysis showed that the expression of *AhR1* displayed linear (*P_lin_* = 0.026) and quadratic (*P_quad_* = 0.004) patterns of decrease with increasing PB inclusion level. At week 12, the expression of *AhR1* (*P* < 0.001), *ARNT* (*P* = 0.003), *CYP1A1* (*P* < 0.001) and *CYP1A2* (*P* = 0.016) differed between the experimental treatments, as shown in Table 4. Additionally, increasing PB inclusion level resulted in linear decrease for *AhR1* (*P_li_*_n_ < 0.001), *ARNT* (*P_lin_* = 0.004), *CYP1A1* (*P_lin_* < 0.001), *CYP1A2* (*P_lin_* = 0.011) and *CYP1B1* (*P_lin_* = 0.039). Furthermore, dietary PB inclusion level resulted in patterns of expression decrease in a quadratic manner for *AhR1* (*P_quad_* < 0.001) and *CYP1A1* (*P_quad_* = 0.011). The relative gene expression of *AhR1* was lowest for treatment PB750 and of *ARNT* for PB1000, compared to control treatment. In the case of *CYP1A1* and CYP1A2, PB1500 treatment had the lowest expression, always compared to CON.

**Table 4.** Relative gene expression levels of the AhR pathway-related genes in layers’ ovary.

	AhR pathway	Treatments^1^						Statistics^2^		
Ovary	Genes^4^	CON	PB250	PB750	PB1000	PB1500	SEM^3^	*P_anova_*	*P_linear_*	*P_quadratic_*
Week 6 (27-week-old)	*AhR1*	1.61	0.93	0.78	0.96	1.02	0.220	0.132	0.026	0.004
	*ARNT*	1.13	1.00	1.03	0.96	0.96	0.168	0.843	0.327	0.729
	*CYP1A1*	1.36	0.90	1.09	1.00	0.87	0.210	0.160	0.072	0.505
	*CYP1A2*	1.10	0.83	1.01	1.12	0.94	0.139	0.228	0.895	0.761
	*CYP1B1*	1.41	0.81	1.13	1.11	0.91	0.215	0.086	0.159	0.410
Week 12 (33-week-old)	*AhR1*	2.33^a^	1.16^b^	0.74^b^	0.80^b^	0.75^b^	0.265	<0.001	<0.001	<0.001
	*ARNT*	1.67^b^	1.72^b^	1.05^ab^	0.70^b^	0.72^b^	0.415	0.003	0.004	0.817
	*CYP1A1*	3.57^a^	1.93^b^	1.09^bc^	0.66^c^	0.36^c^	0.432	<0.001	<0.001	0.011
	*CYP1A2*	2.25^a^	0.87^b^	1.06^bc^	0.98^bc^	0.87^c^	0.440	0.016	0.011	0.061
	*CYP1B1*	1.84	1.24	0.84	0.78	1.04	0.428	0.189	0.039	0.076

^1^PB Inclusion (CON = 0 mg/kg, PB250 = 250mg/kg, PB750 = 750mg/kg, PB1000 = 1000mg/kg and PB1500 = 1500mg/kg of diet). Data represent treatment means based on 1 hen per replicate and 7 replicates per treatment (*n* = 7).

^2^Means with no common superscripts (a, b, c) within the same row differ significantly (*P* < 0.05).

^3^Standard error of the means.

^4^AhR1 = aryl hydrocarbon receptor 1; ARNT = aryl hydrocarbon receptor nuclear translocator; CYP1A1 = cytochrome P450 1A1; CYP1A2 = cytochrome P450 1A2; CYP1B1 = cytochrome P450 1B1.

### mRNA transcript levels of the AhR pathway genes in the liver at the 6th and 12th week

In the liver, the relative expression levels of the *AhR* pathway (*AhR1*, *ARNT*, *CYP1A1*, *CYP1A2* and *CYP1B1*) related genes at week 6 and week 12 (i.e., 27 and 33 week of layers age) are presented in Table 5. At week 6, dietary PB inclusion level, down-regulated the expression of *CYP1A1* (*P* < 0.001) and *CYP1A2* (*P* = 0.002) for all PB treatments, compared to CON. Polynomial contrast analysis revealed that the expression of *CYP1A1* (*P_lin_* < 0.001), *CYP1A2* (*P_lin_* = 0.002) and *CYP1B1* (*P_lin_* = 0.015) displayed a linear pattern of decrease with increasing PB inclusion level. In addition, increasing PB inclusion level resulted in a quadratic pattern of decrease (*P_quad_* < 0.001) for the expression of *CYP1A1* and *CYP1A2.* The relative gene expression of *CYP1A1* was lowest for treatment PB750 and of *CYP1A2* for PB1000, compared to control treatment. At week 12, the expression of *AhR1* (*P* = 0.037), *ARNT* (*P* = 0.010), *CYP1A1* (*P* = 0.020), *CYP1A2* (*P* = 0.003) and *CYP1B1* (*P* = 0.002) differed between the experimental treatments, as shown in Table 5. Additionally, increasing PB inclusion level resulted in linear patterns of decrease (*P_lin_* ≤ 0.05) for all of the AhR pathway genes assessed. Moreover, increasing dietary PB inclusion level decreased in a quadratic manner the expression of *AhR1* (*P_quad_* = 0.045). The relative gene expression of *AhR1* was lowest for treatment PB1000, compared to control treatment. In the case of *ARNT*, *CYP1A1, CYP1A2* and *CYP1B1* the PB1500 treatment had the lowest expression, compared to CON.

**Table 5.** Relative gene expression levels of the AhR pathway-related genes in layers’ liver.

	AhR pathway	Treatments^1^						Statistics^2^		
Liver	Genes^4^	CON	PB250	PB750	PB1000	PB1500	SEM^3^	*P_anova_*	*P_linear_*	*P_quadratic_*
Week 6 (27-week-old)	*AhR1*	1.11	1.11	0.89	0.88	1.01	0.153	0.387	0.216	0.253
	*ARNT*	1.29	1.17	0.97	1.40	1.41	0.224	0.271	0.347	0.142
	*CYP1A1*	2.33^a^	0.90^b^	0.61^b^	0.80^b^	1.11^b^	0.263	<0.001	<0.001	<0.001
	*CYP1A2*	2.10^a^	0.89^b^	0.74^b^	0.65^b^	1.14^b^	0.279	0.002	0.002	<0.001
	*CYP1B1*	1.50	1.03	1.00	0.84	0.91	0.237	0.073	0.015	0.134
Week 12 (33-week-old)	*AhR1*	1.30^a^	1.10^ab^	0.91^ab^	0.82^b^	0.99^ab^	0.153	0.037	0.014	0.045
	*ARNT*	2.20^a^	1.69^ab^	0.96^b^	1.10^ab^	0.84^b^	0.403	0.010	0.001	0.217
	*CYP1A1*	1.74^a^	1.06^ab^	1.18^ab^	0.72^b^	0.61^b^	0.340	0.020	0.002	0.527
	*CYP1A2*	1.78^a^	1.04^ab^	0.98^b^	0.77^b^	0.77^b^	0.258	0.003	<0.001	0.061
	*CYP1B1*	1.48^a^	1.06^ab^	1.16^ab^	0.79^b^	0.77^b^	0.179	0.002	<0.001	0.427

^1^PB Inclusion (CON = 0 mg/kg, PB250 = 250mg/kg, PB750 = 750mg/kg, PB1000 = 1000mg/kg and PB1500 = 1500mg/kg of diet). Data represent treatment means based on 1 hen per replicate and 7 replicates per treatment (*n* = 7).

^2^Means with no common superscripts (a, b) within the same row differ significantly (*P* < 0.05).

^3^Standard error of the means.

^4^AhR1 = aryl hydrocarbon receptor 1; ARNT = aryl hydrocarbon receptor nuclear translocator; CYP1A1 = cytochrome P450 1A1; CYP1A2 = cytochrome P450 1A2; CYP1B1 = cytochrome P450 1B1.

### mRNA transcript levels of the Nrf2 pathway genes in the ovary at the 6th and 12th week

In the ovary, the relative expression levels of the *Keap1/Nrf2/ARE* pathway (*Nrf2*, *Keap1*, *CAT*, *SOD1*, *GPX2*, *GSTA2*, *NQO1* and *HMOX1*) related genes at week 6 and week 12 (i.e., 27 and 33 week of layers age) are presented in Table 6. Significant changes between the experimental treatments were shown for the expression of *Nrf2* (*P* = 0.016), *Keap1* (*P* = 0.011), *CAT* (*P* < 0.001), *GPX2* (*P* = 0.002), *GSTA2* (*P* = 0.030) and *HMOX1* (*P* < 0.001). Polynomial contrast analysis showed that the relative expression of *CAT*, *GPX2* and *HMOX1* displayed linear patterns of increase (*P_lin_* ≤ 0.05). In particular, the relative expression of the genes above displayed quadratic patterns of changes with increasing PB inclusion level. More specifically, the expression of *Nrf2* (*P_quad_* = 0.003), *GPX2* (*P_quad_* < 0.001), *GSTA2* (*P_quad_* = 0.006) and *HMOX1* (*P_quad_* = 0.002) reached the highest levels in treatment PB750. The relative gene expression of *Keap1* and *CAT* was lowest for treatment PB750 and highest for PB1000, respectively, always compared to CON. At week 12, significant differences between the experimental treatments were shown for the expression of *Nrf2* (*P* = 0.029), *Keap1* (*P* = 0.015), *SOD1* (*P* = 0.017), *GPX2* (*P* = 0.017), *GSTA2* (*P* = 0.006), *NQO1* (*P* = 0.012) and *HMOX1* (*P* < 0.001), as shown in Table 6. The expression, of *SOD1, GPX2* and *HMOX1* showed linear (*P_lin_* ≤ 0.05) patterns of increase with increasing PB inclusion level and decrease of *Keap1*. In addition, the relative expression of *Nrf2*, *GSTA2* and *HMOX1* displayed quadratic patterns of increase (*P_quad_* = 0.017, 0.002 and 0.016, respectively) with increasing PB inclusion level. Compared to CON, the relative expression levels were highest for PB250 and PB1500 for the first (i.e., *Nrf2* and *NQO1*) and second (i.e., *SOD1* and *GPX2*) set of genes above, respectively. In the case of *GSTA2* and *HMOX1*, treatment PB750 had the highest expression levels compared to CON and the lowest for *Keap1*.

**Table 6.** Relative gene expression levels of the Nrf2 pathway-related genes in layers’ ovary.

	Nrf2 pathway	Treatments^1^						Statistics^2^		
*Ovary*	Genes^4^	CON	PB250	PB750	PB1000	PB1500	SEM^3^	*P_anova_*	*P_linear_*	*P_quadratic_*
Week 6 (27-week-old)	*Nrf2*	0.82^b^	1.08^ab^	1.38^a^	1.24^ab^	1.04^ab^	0.158	0.016	0.098	0.003
	*KEAP1*	1.31^a^	0.84^b^	0.80^b^	0.94^a^	1.14^a^	0.154	0.011	0.481	0.001
	*CAT*	0.56^b^	1.01^ab^	1.41^a^	1.52^a^	1.43^a^	0.206	<0.001	<0.001	0.019
	*SOD1*	0.98	1.08	1.34	1.23	1.39	0.243	0.418	0.086	0.706
	*GPX2*	0.51^b^	1.29^ab^	1.85^a^	1.38^a^	1.18^ab^	0.287	0.002	0.033	<0.001
	*GSTA2*	0.70^b^	1.21^ab^	1.72^a^	1.41^ab^	1.15^ab^	0.301	0.030	0.113	0.006
	*NQO1*	1.18	1.19	1.26	1.17	1.23	0.213	0.994	0.891	0.900
	*HMOX1*	0.46^c^	1.07^bc^	2.10^a^	1.35^ab^	1.61^ab^	0.279	<0.001	<0.001	0.002
Week 12 (33-week-old)	*Nrf2*	0.61^b^	1.73^a^	1.48^a^	1.71^a^	1.37^ab^	0.366	0.029	0.076	0.017
	*KEAP1*	1.63^a^	1.37^ab^	0.65^b^	0.90^b^	0.79^b^	0.252	0.015	0.001	0.067
	*CAT*	0.56	1.35	1.58	1.28	1.60	0.465	0.185	0.062	0.236
	*SOD1*	0.71^b^	1.40^ab^	1.13^ab^	1.30^ab^	2.12^a^	0.383	0.017	0.004	0.491
	*GPX2*	0.67^b^	1.23^ab^	1.41^ab^	1.46^a^	1.54^a^	0.263	0.017	0.002	0.126
	*GSTA2*	0.64^b^	1.92^a^	1.48^a^	1.37^ab^	1.02^ab^	0.321	0.006	0.773	0.002
	*NQO1*	0.89^b^	1.73^a^	1.16^ab^	0.98^b^	1.19^ab^	0.237	0.012	0.779	0.180
	*HMOX1*	0.53^b^	1.77^a^	1.84^a^	1.20^ab^	1.63^a^	0.345	<0.001	0.043	0.016

^1^PB Inclusion (CON = 0 mg/kg, PB250 = 250mg/kg, PB750 = 750mg/kg, PB1000 = 1000mg/kg and PB1500 = 1500mg/kg of diet). Data represent treatment means based on 1 hen per replicate and 7 replicates per treatment (*n* = 7).

^2^Means with no common superscripts (a, b, c) within the same row differ significantly (*P* < 0.05).

^3^Standard error of the means.

^4^Nrf2 =nuclear factor; erythroid 2-like 2; Keap1 = kelch-like ECH-associated protein 1; CAT = catalase; SOD1 =superoxide dismutase 1; GPX2 =glutathione peroxidase 2; GSTA2 =glutathione S-transferase alpha 2; NQO1 = NADPH quinone dehydrogenase 1; HMOX1 = heme oxygenase 1.

### mRNA transcript levels of the Nrf2 pathway genes in the liver at the 6th and 12th week

In the liver, the relative expression levels of the *Keap1/Nrf2/ARE* pathway (*Nrf2*, *Keap1*, *CAT*, *SOD1*, *GPX2*, *GSTA2*, *NQO1* and *HMOX1*) related genes at week 6 and week 12 (i.e., 27 and 33 week of layers age) are presented in Table 7. Significant changes between the experimental treatments were shown for the expression of *Nrf2* (*P* = 0.030), *Keap1* (*P* = 0.001), *CAT* (*P* = 0.001), *GSTA2* (*P* = 0.012) and *NQO1* (*P* = 0.021). Polynomial contrast analysis showed that the relative expression of Nrf2, *Keap1* and *CAT* displayed linear patterns of change (*P_lin_* ≤ 0.05). In particular, the relative expression of *Keap1, SOD1, GSTA2* and *HMOX1* displayed quadratic patterns of changes with increasing PB inclusion level. More specifically, the expression of *Keap1* (*P_quad_* = 0.049) and *GSTA2* (*P_quad_* = 0.001), reached the lowest and highest levels, respectively, in treatment PB750. The relative gene expression of *SOD1* and *HMOX1* was highest for treatment PB250, always compared to CON. At week 12, significant differences between the experimental treatments were shown for the expression of *CAT* (*P* = 0.023), *SOD1* (*P* = 0.012 and *NQO1* (*P* < 0.001), as shown in Table 7. The relative expression of SOD1 and *NQO1* displayed quadratic patterns of increase (*P_quad_* = 0.024 and 0.003, respectively) with increasing PB inclusion level. Compared to CON, the relative expression of *CAT* was highest for PB1500. In the case of *SOD1* and *NQO1*, treatment PB750 had the highest expression levels compared to CON.Paragraph: use this for the first paragraph in a section, or to continue after an extract.

**Table 7.** Relative gene expression levels of the Nrf2 pathway-related genes in layers’ liver.

	Nrf2 pathway	Treatments^1^						Statistics^2^		
Liver	Genes^4^	CON	PB250	PB750	PB1000	PB1500	SEM^3^	*P_anova_*	*P_linear_*	*P_quadratic_*
Week 6 (27-week-old)	*Nrf2*	0.67^b^	1.22^ab^	1.59^a^	1.61^a^	1.61^a^	0.329	0.030	0.004	0.101
	*KEAP1*	1.25^a^	1.00^ab^	0.85^b^	0.96^b^	0.86^b^	0.095	0.001	<0.001	0.049
	*CAT*	0.66^b^	1.15^ab^	1.45^a^	1.24^a^	1.48^a^	0.195	0.001	<0.001	0.058
	*SOD1*	0.73	1.46	1.35	1.24	1.23	0.265	0.090	0.208	0.043
	*GPX2*	1.00	1.16	1.43	1.06	1.46	0.342	0.556	0.288	0.857
	*GSTA2*	0.80^b^	1.29^ab^	1.70^a^	1.35^ab^	0.88^b^	0.266	0.012	0.706	0.001
	*NQO1*	0.81^b^	1.35^a^	1.12^ab^	1.13^ab^	1.18^ab^	0.150	0.021	0.126	0.075
	*HMOX1*	0.76	1.41	1.27	1.22	1.19	0.235	0.101	0.215	0.050
Week 12 (33-week-old)	*Nrf2*	1.10	1.33	1.29	0.87	1.04	0.202	0.158	0.197	0.350
	*KEAP1*	1.16	0.93	1.17	0.77	0.84	0.219	0.258	0.115	0.957
	*CAT*	0.77^b^	1.40^ab^	1.30^ab^	0.96^ab^	1.43^a^	0.226	0.023	0.090	0.353
	*SOD1*	0.70^b^	1.39^a^	1.47^a^	1.04^ab^	1.28^ab^	0.223	0.012	0.114	0.024
	*GPX2*	1.09	0.59	1.35	1.44	1.38	0.349	0.116	0.076	0.834
	*GSTA2*	0.78	1.32	1.33	1.31	1.20	0.349	0.127	0.141	0.096
	*NQO1*	1.07^b^	1.22^ab^	1.51^a^	0.91^b^	0.99^b^	0.122	<0.001	0.104	0.003
	*HMOX1*	1.48	1.88	1.73	1.43	1.36	0.556	0.866	0.576	0.471

^1^PB Inclusion (CON = 0 mg/kg, PB250 = 250mg/kg, PB750 = 750mg/kg, PB1000 = 1000mg/kg and PB1500 = 1500mg/kg of diet). Data represent treatment means based on 1 hen per replicate and 7 replicates per treatment (n = 7).

^2^Means with no common superscripts (a, b) within the same row differ significantly (P < 0.05).

^3^Standard error of the means.

^4^Nrf2 =nuclear factor; erythroid 2-like 2; Keap1 = kelch-like ECH-associated protein 1; CAT = catalase; SOD1 =superoxide dismutase 1; GPX2 =glutathione peroxidase 2; GSTA2 =glutathione S-transferase alpha 2; NQO1 = NADPH quinone dehydrogenase 1; HMOX1 = heme oxygenase 1.

## Discussion

This work has followed a nutrigenomic approach to generate new information on the effects of a Mediterranean plant-based dietary phytogenic blend on critical physiological cytoprotective responses in the ovary and liver of young laying hens. Thus, critical gene components of two essential homeostasis pathways related to detoxification (AhR) and antioxidant response (Nrf2), briefly discussed below, were monitored at two discrete time phases (i.e., 6^th^ and 12^th^ experimental week). Furthermore, layer performance responses were monitored daily during the experiment.

Poultry zootechnical performance is associated with overall ovary and liver health. Upon the aging process of the breeder and laying hens, various organs such as the ovary and liver experience a decrease in functionality, accompanied by reduced antioxidant capacity, due to oxidative stress.^2^^,^^17^^,^^22^^,^^25^ Ovary and liver dysfunction in poultry can have several adverse effects, such as impaired reproductive performance, poor egg quality and hatching rate, as well as leading to metabolic disorders.^26–28^ These issues could be prevented and/or alleviated via nutritional interventions using suitable bioactive feed additives, ensuring the well-being and long-term productivity of laying hens. The protective role of the dietary Mediterranean indigenous herbs and spices is reflected by their health-promoting properties, attributed to the multivarious bioactive components that they contain.^8–10^ In particular, potential inducers such as phytogenic compounds could shield the host against oxidative damage, by activating two critical pathways, namely aryl hydrocarbon receptor (AhR) and nuclear factor erythroid 2-related factor 2 (Nrf2), and their downstream genes with detoxifying, antioxidant and anti-inflammatory properties.^14–16^^,^^29^

The AhR is a critical transcription factor related to detoxification and its activation is ligand-mediated.^30^^,^^31^ AhR ligands (e.g., mycotoxins, environmental toxicants, phytochemical compounds and bacterial pathogens) bind to the AhRs multiprotein complex and get translocated into the nucleus. After heterodimerization with the AhR nuclear translocator (*ARNT*), the AhR-ARNT complex binds to the xenobiotic responsive element (XRE), regulating the transcription of the xenobiotic-metabolizing enzymes (XME), referred to as phase I enzymes. Moreover, the cytochrome P450 (CYP) enzymes (e.g., *CYP1A1, CYP1A2, CYP1B1*) participate in Phase I metabolism involved in the detoxification of xenobiotics.^16^^,^^30^^,^^31^

Widely regarded as the key regulator of the cellular antioxidant response, Nrf2 is a vital transcription factor for host protection against oxidative stress and damage.^12^^,^^32^^,^^33^ Under physiological conditions, the Nrf2 remains bound to the Kelch-like ECH associating protein 1 (Keap1) maintaining its negative regulation within the cytoplasm.^14^^,^^32^ Several natural compounds (e.g., phytogenics) disrupt the Nrf2-Keap1 cytoplasmic complex, allowing the nuclear translocation of Nrf2. In the nucleus Nrf2 binds to the antioxidant response element (ARE) and controls the transcription of multifarious cytoprotective enzymes known for their antioxidant (e.g., *CAT, SOD1, GPX2*), detoxifying (e.g., *GSTA2, NQO1*) and anti-inflammatory (e.g., *HMOX1*) properties.^14^^,^^16^^,^^29^^,^^34^

In this study, the main experimental trial lasted for 12 weeks and started when the laying hens were at the beginning of the peak production period (i.e., 22–33 week of layers age). The zootechnical performance results showed that the PB supplementation in layers diets significantly improved the overall laying rate, egg mass and feed conversion ratio. Regarding the performance results, the optimal PB inclusion level for enhancing the laying productive performance was 750 mg/kg of diet. Generally, the findings of this study with respect to performance benefits were in line with other studies assessing Mediterranean plant-derived dietary supplements and reported improved layer productive performance. Example cases include rosemary powder,^35^ thyme,^36^ oregano essential oil,^37^ olive leaf extract^38^ and a phytogenic premix derived from ginger, lemon balm, oregano, and thyme.^39^ These performance benefits could be attributed to the phytogenic content of bioactive compounds, which have been shown to possess cytoprotective and anti-inflammatory functions.

In addition to the zootechnical performance improvements, PB inclusion level beneficially modulated the AhR pathway genes in the ovary and liver of laying hens. At the 6^th^ week of the experiment (i.e., layers 27 week-old), significant changes (*CYP1A1* and *CYP1A2*) were seen only in the liver. However, after 6 weeks of high productivity (i.e., 12^th^ experimental week—layers 33 week-old) the PB inclusion significantly down-regulated the majority (4 out of 5) of the AhR pathway genes studied in the ovary, as well as all of the AhR pathway genes studied in the liver. According to Antos et al. (2015)^40^, the primary organ responsible for detoxification is the liver, with the ovary playing a subsidiary role in this process. The noted down-regulation of the AhR-related gene expression during the peak production phase may suggest decreased detoxification demands, attributed to the PB inclusion. Besides the detoxification process, the AhR plays a crucial role in regulating various physiological processes, including reproduction and its activation can intensify age-related changes in the ovary and overall reproductive function. In particular, AhR activation accelerates reproductive senescence, possibly by disrupting ovarian and hypothalamic function.^41–43^

In view of the above, we could hypothesize an indirect positive impact on the layer/breeder hen reproductive process resulting from the downregulation of the AhR pathway through the inclusion of PB. With respect to breeder production and management, the decreased detoxification requirements may suggest a reduced level of xenobiotics in the ovary, indicating a more secure environment for the developing embryo but this will have to be specifically studied in future studies.

In this study, the PB inclusion beneficially modulated the expression of the transcription factor Nrf2 and its downstream genes. At the 6^th^ experimental week (i.e., layers 27 week-old), the majority of the Nrf2 pathway genes studied significantly responded to PB inclusion level in the layer’s ovary (6 out of 8 genes) and the liver (5 out of 8 genes). The priming of antioxidant responses by the phytogenic blend used in this work was also demonstrated at the 12^th^ experimental week (i.e., layers 33 week-old). In particular, beneficial modulation of the Nrf2 pathway genes was seen in the ovary (7 out of 8 genes) and liver (3 out of 8 genes). Indicatively, the PB inclusion up-regulated the mRNA levels of several Phase II enzymes, also known as ‘indirect antioxidant enzymes’ (i.e., *Nrf2, CAT, SOD1, GPX2, GSTA2, NQO1, HMOX1*) while down-regulated the Keap1 which serves as an inhibitor of *Nrf2*. The above ovarian and hepatic transcriptomic results demonstrate the PB priming of the layer protective potential against arising threats linked to oxidative stress. The latter contributes to ovarian aging and age-related disorders, as well as is considered a key factor contributing to lipid accumulation and liver injury through complex signaling pathways.^21^^,^^25^^,^^44–46^

The prompt and adequate control of ROS and subsequent oxidative stress is a prerequisite for sustaining both egg production and the overall health of the birds.^28^^,^^36^^,^^47^ The above is of particular importance during the pre-peak/peak production phase which can have long-lasting beneficial effects until the late-phase of production, such as prolonging the female reproductive lifespan and persistence of egg production.^19^^,^^22^^,^^48^ In this sense, our findings revealed the cytoprotective potential of PB inclusion level that can safeguard birds from various stressors, ensuring cellular homeostasis and functionality.

This study followed a PB dose response to identify the most optimal inclusion level with respect to layer production performance and critical biological cytoprotective responses. Indeed, the study results showed that the effects of dietary PB on zootechnical performance and the gene expression responses were dose dependent. Regarding the performance responses, increasing the PB inclusion level from 250 to 1500 mg/kg of diet resulted in a linear and quadratic manner of change pointing to 750 mg PB/kg of diet as the most optimal. Furthermore, ovarian/hepatic nutrigenomic analysis at the ovarian/hepatic level, highlighted PB beneficial modulatory effects in the expression of the cytoprotective genes with PB inclusion levels from 750 to 1500 mg/kg diet being the most effective. Therefore, under non-challenge experimental conditions, the PB inclusion level of 750 mg/kg diet seems to be the most prominent.

In this work, the dietary PB positively influenced two key pathways (i.e., AhR, Nrf2) essential for the maintenance of homeostasis, suggesting that with improved host functioning more energy and nutrients could be made available for production purposes. Considering all the above together, PB dietary inclusion has resulted in a consistent beneficial modulation of the AhR/Nrf2 pathway-related genes providing a mechanistic basis to further support the zootechnical improvements evidenced in the productivity of the layers.

## Conclusion

The analytical nutrigenomics approach of the present study provided significant mechanistic evidence for the phytogenic function at ovarian and hepatic level in laying hens. It was shown that dietary PB supplementation beneficially modulated the mRNA levels of cytoprotective genes by down-regulating the AhR pathway and up-regulating the Nrf2 ones indicating reduced requirements for detoxification and an increased antioxidant capacity. The latter findings advocate for the productive performance improvements seen, with PB750 displaying the optimal benefits. The study has generated new knowledge that supports zootechnical performance that remains to be further validated under various stressor challenges such as heat and aging.
